# Single-cell RNA sequencing to dissect the immunological network of liver fibrosis in *Schistosoma japonicum*-infected mice

**DOI:** 10.3389/fimmu.2022.980872

**Published:** 2022-12-22

**Authors:** Yu Zhang, Junhui Li, Hao Li, Jie Jiang, Chen Guo, Chen Zhou, Zhaoqin Zhou, Yingzi Ming

**Affiliations:** ^1^ Transplantation Center, The Third Xiangya Hospital, Central South University, Changsha, Hunan, China; ^2^ Engineering and Technology Research Center for Transplantation Medicine of National Health Comission, The Third Xiangya Hospital, Central South University, Changsha, Hunan, China

**Keywords:** *Schistosomiasis japonica*, hepatic fibrosis, single-cell RNA sequencing (scRNAseq), landscape, immune cells

## Abstract

**Introduction:**

Liver fibrosis is a poor outcome of patients with schistosomiasis, impacting the quality of life and even survival. Eggs deposited in the liver were the main pathogenic factors of hepatic fibrosis in Schistosomiasis japonica. However, the mechanism of hepatic fibrosis in schistosomiasis remains not well defined and there is no effective measure to prevent and treat schistosome-induced hepatic fibrosis.

**Methods:**

In this study, we applied single-cell sequencing to primarily explore the mechanism of hepatic fibrosis in murine schistosomiasis japonica (n=1) and normal mouse was served as control (n=1).

**Results:**

A total of 10,403 cells were included in our analysis and grouped into 18 major cell clusters. Th2 cells and NKT cells were obviously increased and there was a close communication between NKT cells and FASLG signaling pathway. Flow cytometry analysis indicated that the expression of Fasl in NKT cells, CD8+ T cell and NK cell were higher in SJ groups. *Arg1*, *Retnla* and *Chil3*, marker genes of alternatively activated macrophages (M2), were mainly expressed in mononuclear phagocyte(1) (MP(1)), suggesting that Kupffer cells might undergo M2-like polarization in fibrotic liver of schistosomiasis. CXCL and CCL signaling pathway analysis with CellChat showed that Cxcl16-Cxcr6, Ccl6-Ccr2 and Ccl5-Ccr5 were the most dominant L−R and there were close interactions between T cells and MPs.

**Conclusion:**

Our research profiled a preliminary immunological network of hepatic fibrosis in murine schistosomiasis japonica, which might contribute to a better understanding of the mechanisms of liver fibrosis in schistosomiasis. NKT cells and CXCL and CCL signaling pathway such as Cxcl16-Cxcr6, Ccl6-Ccr2 and Ccl5-Ccr5 might be potential targets to alleviate hepatic fibrosis of schistosomiasis.

## Introduction

Schistosomiasis, a neglected tropical disease, is caused by infection with worms of the trematode genus *Schistosoma* and affects over 200 million people (1). Three species, *Schistosoma mansoni*, *S. haematobium* and *S. japonicum* are the main disease-causing species. *Schistosoma mansoni* and *Schistosoma japonicum* cause hepatointestinal disease, while *Schistosoma haematobium* is involved in lesions of urologic system (2). Schistosome eggs trapped in host tissues are the main pathogenic factor of schistosomiasis and the host reactions to *S. japonicum* eggs induce formation of granulomas and hepatic fibrosis, which lead to portal hypertension and is the primary cause of morbidity and mortality for schistosomiasis (3).

Schistosome-associated hepatic fibrosis is an immunopathogenic disorder, causing activation of the hepatic stellate cells (HSC) and excessive deposition of extracellular matrix (ECM) components (4, 5). The interactions are complex and diverse between Schistosome-associated hepatic fibrosis and immune cells. Egg antigens promote strongly Th2-mediated inflammatory reactions, which produce a large amount of Th2 cytokines including interleukin (IL)-4, IL-5 and IL-13 and suppresses Th1-mediated response (6). IL-13, a profibrotic cytokines, is capable of directly driving HSC activation through TGF-β-independent signal. Recent studies also demonstrated that Tfh cells, Th17 cell and Th9 cells promote granulomatous inflammation and hepatic fibrosis in schistosomiasis (7–9). Meanwhile, Treg cell, an important regulator, can prevent excessive immunopathogenic response during the development of schistosomiasis, which exhibited a potential role to suppress schistosome‐associated liver fibrosis (10). Macrophages are conventionally classified as classically activated macrophages (M1) and alternatively activated macrophages (M2) (11). After induced by eggs, resident macrophages secrete inflammatory cytokines and chemokines to stimulate the influx of lymphocytes, neutrophils, and monocytes, which initiates granulomatous inflammation (12). And Th2-type cytokines(IL-4 and IL-13) promote M2 polarization, which induce the expression of arginase-1 (Arg-1), Ym-1, and Fizz-1 (13). Arg1-expressing macrophages exhibit anti-fibrotic activity following *S. mansoni* infection through rapid metabolism of L-arginine, a substrate required during the proliferation of lymphocytes (14). Liver sinusoidal endothelial cells (LSECs), presenting antigen and facilitating the adhesion of leukocytes and lymphocytes, might also participate in progression of hepatic fibrosis in schistosomiasis (5, 15). Various cells participate in complex pathogenetic mechanisms of schistosome‐associated liver fibrosis. However, there is no available drugs to prevent the progression or alleviate schistosome-associated hepatic fibrosis, as praziquantel is still the only effective drug being used to treat adult worms of schistosome (16). A better understanding of pathogenesis could lead to improved resolution of schistosome‐associated liver fibrosis.

Single cell RNA-sequencing analyzing the transcriptomes of individual cells is a powerful technology to investigate cellular heterogeneity, which promote advances in disease research (17–21). To better understand the mechanism of schistosomiasis liver fibrosis, we performed single-cell sequencing on fibrotic liver of murine schistosomiasis japonica and verified with RT-PCR and flow cytometry. Revealing the features of immune cells in schistosome‐associated liver fibrosis will help to further clarify the pathogenesis and identify potential targets for treatment of hepatic fibrosis in schistosomiasis.

## Methods

### Hepatic fibrosis mice model of *Schistosomiasis japonica*

Male C57BL/6 mice (6 weeks old) were purchased from Hunan Slack Jingda Experimental Animal Company. Mice with free accessed to food and water were housed under specific-pathogen–free conditions at the Experimental Animal Center of Central South University. The protocols for the animal experiments were approved by the local ethics committee for the use of animals at Central South University (Changsha, China). S. japonicum-infected Oncomelania hupensis snails was provided by Hunan Provincial Institute of Parasitic Diseases in China. After inducing the release of cercariae, mice subsequently infected percutaneously with 25 ± 2 freshly shed cercaria. At day 35 post-infection, the mice were treated with praziquantel (500 mg/kg, once per day for 2 days) using intragastric gavage (22). The mice were sacrificed until week 11 after infection.

### Liver dissociation and preparation

The livers obtained from fibrotic liver of S. japonicum-induced mouse and healthy liver of normal mouse. The fresh tissues were stored in the sCelLive™ Tissue Preservation Solution (Singleron) on ice as quickly as possible. After washed with Hanks Balanced Salt Solution (HBSS) for three times, the specimens were minced into small pieces. To dissociated into single-cell suspensions, the specimens then digested with 3 mL sCelLive™ Tissue Dissociation Solution (Singleron) by Singleron PythoN™ Tissue Dissociation System at 37°C for 15 min. After filtered through a 40-micron sterile strainer, the cell was added the GEXSCOPE^®^ red blood cell lysis buffer (RCLB, Singleron), which was incubated at room temperature for 5-8 min to remove red blood cells. Centrifugation was performed at 300 × g 4°C for 5 mins to remove supernatant and single cells were resuspended softly in PBS. Finally, the cell viability was evaluated microscopically through staining with Trypan Blue.

### RT & amplification & library construction

Single-cell suspensions (2×10^5^ cells/mL) with PBS (HyClone) were loaded onto microwell chip using the Singleron Matrix^®^ Single Cell Processing System. Barcoding Beads are subsequently collected from the microwell chip, followed by reverse transcription of the mRNA captured by the Barcoding Beads and to obtain cDNA, and PCR amplification. The amplified cDNA is then fragmented and ligated with sequencing adapters. The scRNA-seq libraries were constructed according to the protocol of the GEXSCOPE^®^ Single Cell RNA Library Kits (Singleron) (23). Individual libraries were diluted to 4 nM, pooled, and sequenced on Illumina novaseq 6000 with 150 bp paired end reads.

### Primary analysis of raw read data

Raw reads from scRNA-seq were processed to generate gene expression matrixes using CeleScope (https://github.com/singleron-RD/CeleScope) v1.9.0 pipeline. Briefly, raw reads were first processed with CeleScope to remove low quality reads with Cutadapt v1.17 to trim poly-A tail and adapter sequences (24). Cell barcode and UMI were extracted. After that, we used STAR v2.6.1a to map reads to the reference genome GRCm38 (ensembl version 92 annotation) (24). UMI counts and gene counts of each cell were acquired with featureCounts v2.0.1 software, and used to generate expression matrix files for subsequent analysis (25).

### scRNA-seq quantifications and analysis

RNA-Sequencing data were analyzed such as cell type identification and clustering analysis with the Seurat program (https://satijalab.org/seurat/) (26, 27).Unique molecular identifier (UMI) count tables were loaded into R (R version 4.0.2) using the read.table function. To identify differentially expressed genes (DEGs), we used the Seurat FindMarkers function based on Wilcox likelihood-ratio test with default parameters, and selected the genes expressed in more than 10% of the cells in a cluster and with an average log(Fold Change) value greater than 0.25 as DEGs. KEGG functional enrichment analysis was performed on DEGs to reveal pathways that were significantly associated with the genes specifically expressed (28). The cell-cell interaction analysis was performed by CellChat based on known the interactions between signaling ligands, receptors, and their cofactors (29). Upon inferring the intercellular communication network, CellChat provides functionality for further data exploration, analysis, and visualization.

### RNA extraction and RT-PCR assays

According to the manufacturer’s instruction, total RNA was extracted using SteadyPure Quick RNA Extraction Kit (Accurate biology, China). Real-time polymerase chain reaction (RT-PCR) was performed on the Roche LightCycler^®^ 480 II using gene-specific primers and Universal SYBR Green Master Mix (ABdonal, China). Relative mRNA ratio was used to analyze the data and GAPDH was used as the internal reference. The primer sequence was presented in **Supplementary Table 1**.

### Flow cytometry

After obtain single cells of perfused liver, the cells were incubated with Fixable Viability Dye(BioLegend, San Diego, CA, USA) to distinguish between dead and living cells. After application of the FcR Blocking Reagent (Miltenyi Biotec, Bergisch Gladbach, Germany), single-cell suspensions were stained with the following fluorochrome-conjugated antibodies including CD45-Super bright 600 (eBioscience, San Diego, CA, USA), CD3-FITC, CD4-PerCP-Cy5.5, CD8-PE-Cy7, NK1.1-BV421, CD19-APC-Cy7 and FasL-APC (the rest of antibodies were BioLegend). Flow cytometry was performed using a BD FACS Canto II, and the results were analyzed using FlowJo 10.4 software (Tree Star, Ashland, OR, USA).

### Statistical analysis

The significance level was tested by unpaired t test and all the data are expressed as the means ± s.d. P-values < 0.05 were considered statistically significant. The calculations were performed using GraphPad Prism software package 8.0 (GraphPad Prism, San Diego, CA, USA).

## Results

### Single-cell RNA sequencing and atlas of *Schistosoma japonicum*‐associated liver fibrosis

**Figure 1:**
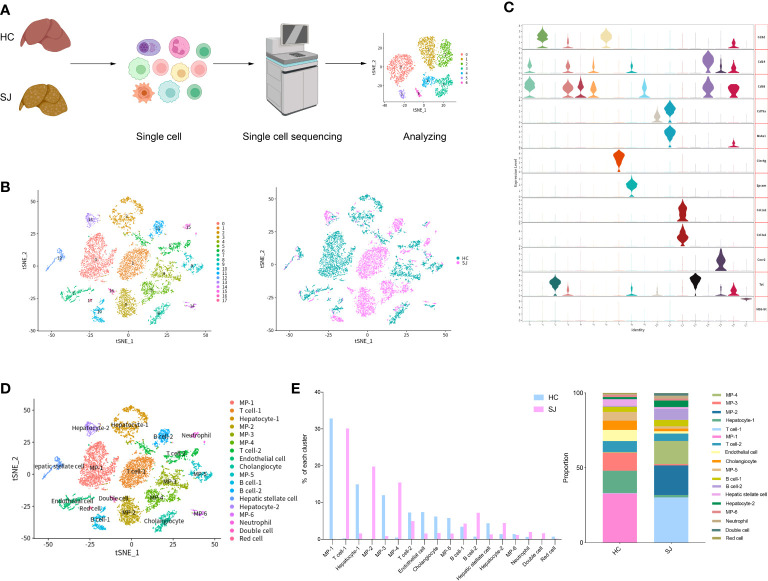
Overview of the 10,403 single cells isolated from liver of S. japonicum-induced mouse and normal mouse. **(A)** The flowchart of our study including grouping, dissociating into single cell, sequencing and analyzing. **(B)** tSNE plot of different clusters and two groups [S. japonicum-induced mouse (SJ, n=1), health control mouse (HC, n=1)]. **(C)** Expression of marker genes in different clusters. **(D)** tSNE plot of annotation clusters. **(E)** The percentage change tendency of each cell cluster in two groups.

We constructed *S. japonicum*-induced mouse models for hepatic fibrosis and mouse livers were obtained at week 11 after *S. japonicum* infection. Single cells were dissociated from a fibrotic liver of *S. japonicum*-infected mouse(SJ, n=1) and a normal mouse was served as control(HC, n=1). After single cells were subjected to single-cell sequencing, we performed further biological analysis on the resultant data(**Figure 1A**). After performing quality control analyses, we filtered out cells with unique feature counts < 200 or > 10 000, and included cells containing less than 25% of mitochondrial genes. Further analysis was conducted on a total of 10,403 cells including 5824 cells from normal liver and 4579 cells from fibrotic livers. Principal component analysis (PCA) was subjected on the top 4,000 variable genes, and the first 15 Principal Components (PCs) were used to calculate clusters with a resolution of 0.3 for clustering analysis, using the Seurat function “FindClusters”. The tSNE plot showed 18 major cell clusters and also presented the distribution of different group(*S. japonicum*-infected mouse SJ, health control mouse HC) (**Figure 1B**). We annotated all of clusters according violin plots of the marker genes and identified mononuclear phagocytes (MP, 6 cell clusters, Cd68+Cd79a-CD3d- and Cd14+Cd79a-CD3d-), T cells (2 cell clusters, Cd3d+Cd68-Cd14-Cd79a-), B cells (2 cell clusters, Cd79a+CD3d-CD68-CD14- and Ms4a1+CD3d-CD68-CD14-), neutrophils (1 cell clusters, Cxcr2+Cd3d-Cd68-Cd79a-), hepatic stellate cell (1 cell clusters, Col3a1+Col1a2+Clec4g-Epcam-), endothelial cell (1 cell clusters, Clec4g+Col3a1-Col1a2-Epcam-), cholangiocyte (1 cell clusters, Epcam+Clec4g-Col3a1-Col1a2-), hepatocyte(2 cell clusters, Tat+Epcam-Clec4g-Hbb-bt-), red cell (1 cell clusters, Hbb-bt+Tat-Epcam-Clec4g-) and Double cell (1 cell clusters, Cd3d+Cd14+Cd68+Tat+Ms4a1) (**Figures 1C, D**). There was obvious difference between SJ group and HC group according to the percentage of different clusters. The clusters of SJ group were composed predominantly of T cell-1,MP-2 and MP-4, while MP-1, MP-3 and Hepatocyte-1 dominated in HC group (**Figure 1E**).

### Increased Th2 cells and NKT cells in fibrotic liver of *Schistosomiasis japonica*

**Figure 2:**
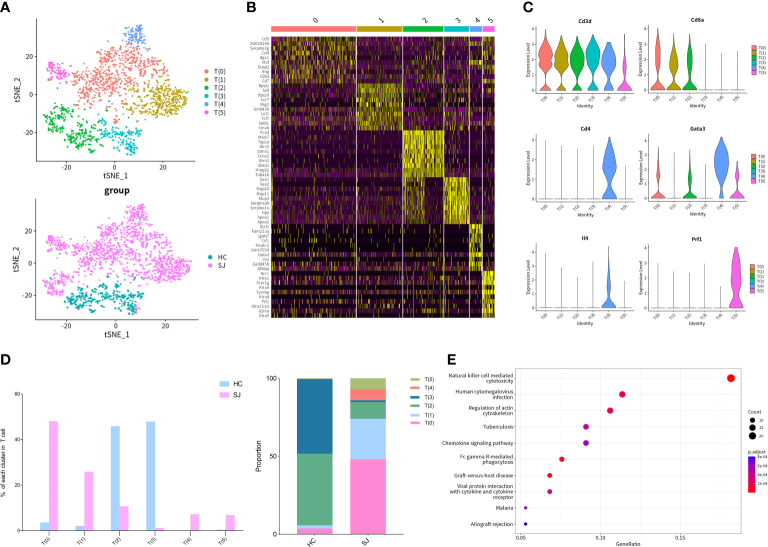
Th2 cells and NKT cells in fibrotic liver of schistosomiasis japonica. **(A)** tSNE plots of the 2,045 T cells for six clusters and two groups. **(B)** Heatmap of T cells for the top 10 DEGs of each cluster. **(C)** Violin plots of genes in each T cell cluster. **(D)** The percentage change tendency and contribution of each T cell cluster in two groups. **(E)** Bubble plots of KEGG pathway enrichment DEGs between T(5) and other T subsets.

We detected 2,045 T cells which were clustered into 6 main clusters and tSNE plot showed that there were great differences in distribution between SJ group and HC group (**Figure 2A**). The heatmap showed the top 10 DEGs of each T cells cluster (**Figure 2B**). All clusters expressed Cd3d, which was the classical marker gene of T cells. *Cd8a*, a marker gene of cytotoxic T cells, was presented in T(0), T(1) and T(2) and *Cd4*, a helper T-cell marker, was predominantly expressed in T(4). *Gata3* and *Il4* were also mainly detected in T(4), while *Prf1* was presented in T(5) (**Figure 2C**). The SJ group was mainly consisted of T(0), T(1), T(2), T(4) and T(5), while the HC group was mostly comprised of T(2) and T(3). It was apparent that T(4) and T(5) were only existed in the SJ group (**Figure 2D**). KEGG pathway analysis was performed and the DEGs between T(5) and other T cell subsets were associated to natural killer cell-mediated cytotoxicity and chemokine signaling pathway (**Figure 2E**).

### M2-like polarization of macrophages in fibrotic liver of *Schistosomiasis japonica*

**Figure 3:**
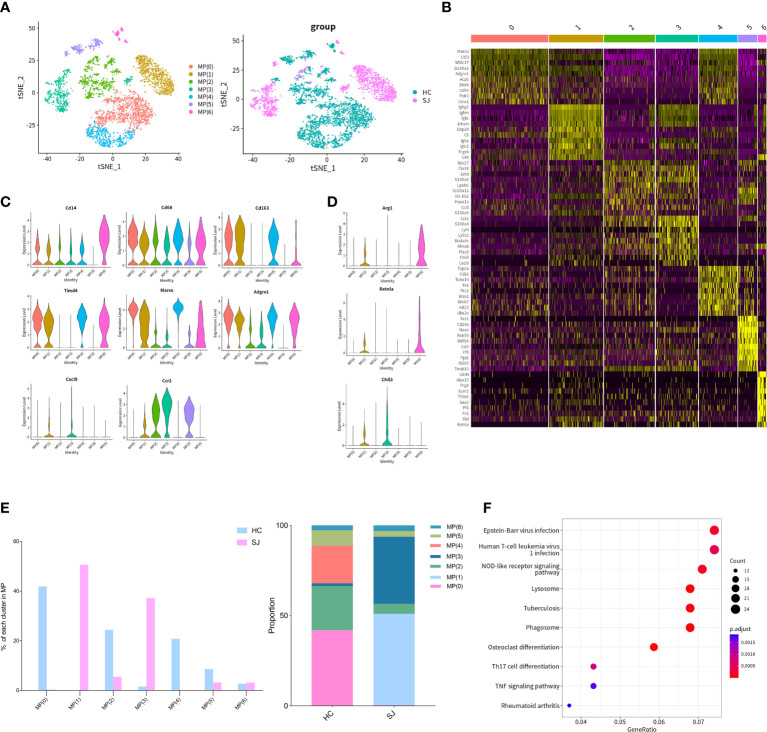
M2-like polarization of macrophages in fibrotic liver of schistosomiasis japonica. **(A)** tSNE plots of the 4,846 monocyte for seven clusters and two groups. **(B)** Heatmap of MPs for the top 10 DEGs of each cluster. **(C)** Violin plots of genes in each MPs cluster. **(D)** Violin plots of M2 maker genes. **(E)** The percentage change tendency and contribution of each MPs cluster in two groups. **(F)** Bubble plots of KEGG pathway enrichment DEGs between MP(1) and other MP subsets.

We detected 4,846 mononuclear phagocytes, which were grouped into 7 clusters. tSNE plot showed that the distribution of clusters was obviously different between SJ group and HC group (**Figure 3A**). Heatmap was used to show the top 10 DEGs of each MPs cluster (**Figure 3B**). *Cd14* was expressed in all MPs clusters but not MP(5) and *CD68* was shown in all MPs clusters. Cd14 and CD68 are surface markers for mononuclear phagocytes. *Cd163*, *Marco*, *Timd4* and *Adgre1*, which were the markers of Kupffer cells, were predominantly expressed in MP(0), MP(1), MP(4) and MP(6) (17). *CCR2* was mainly expressed in MP(1), MP(2), MP(3) and MP(5) and *Cxcl9* was only shown in MP(1) and MP(3) (**Figure 3C**). MP(1) also had high expression of *Arg1*, *Retnla* and *Chil3*, which were the marker genes of alternatively activated macrophages (**Figure 3D**). The SJ group was largely composed of MP(1) and MP(3), while the HC group was mainly consisted of MP(0), MP(2), MP(4) and MP(5) (**Figure 3E**). The KEGG pathways about the DEGs between MP(1) and other MPs subsets were related to NOD-like receptor signaling pathway, lysosome and phagosome (**Figure 3F**).

### Differential enrichment of heterogeneous B cells in fibrotic liver of *Schistosoma japonica*

**Figure 4:**
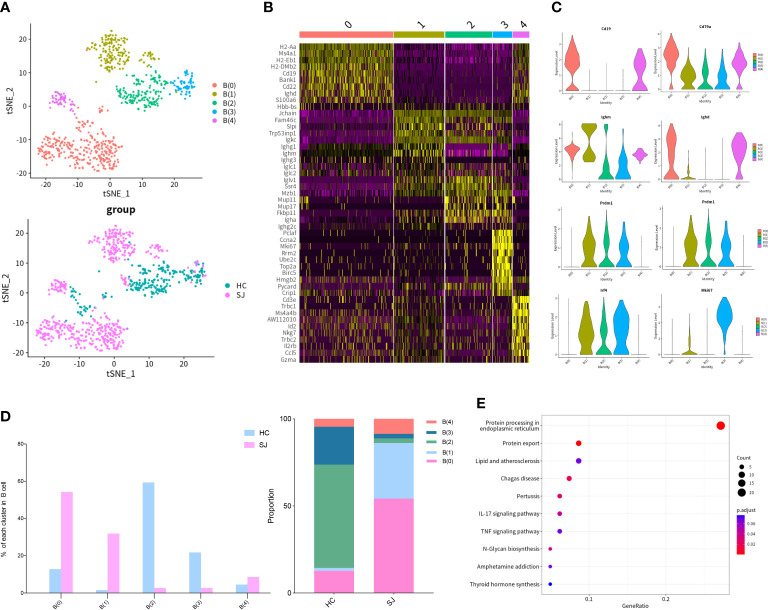
B cells in fibrotic liver of schistosomiasis japonica. **(A)** tSNE plots of the 769 B cells for five clusters and two groups. **(B)** Heatmap of B cells for the top 10 DEGs of each cluster. **(C)** Violin plots of genes in each B cells cluster. **(D)**The percentage change tendency and contribution of each B cells cluster in two groups. **(E)** Bubble plots of KEGG pathway enrichment DEGs between B(1) and other B cells cluster.

We detected 769 B cells which were grouped into 5 clusters and tSNE plot showed different distribution for SJ group and HC group (**Figure 4A**). The heatmap showed the top 10 DEGs of each B cells cluster (**Figure 4B**). *Ighm* and *Cd79a* were expressed in all cluster, while *Cd19* and *Ighd* were expressed predominantly in B(0) and B(4). B(1), B(2) and B(3) were defined as plasmablast with high expression of *Prdm1*, *Sdc1* and *Irf4 (* 30). *Mki67* was presented in B(1) and B(3) (**Figure 4C**). B(0) and B(1) were mainly observed in the SJ group, while the HC group was largely composed of B(0), B(2) and B(3) (**Figure 4D**). The enriched KEGG pathways about the DEGs between B(1) and other B cells cluster were related with protein processing in endoplasmic reticulum and protein export (**Figure 4E**).

### CXCL and CCL signaling pathway network in fibrotic liver of *Schistosoma japonica*

**Figure 5:**
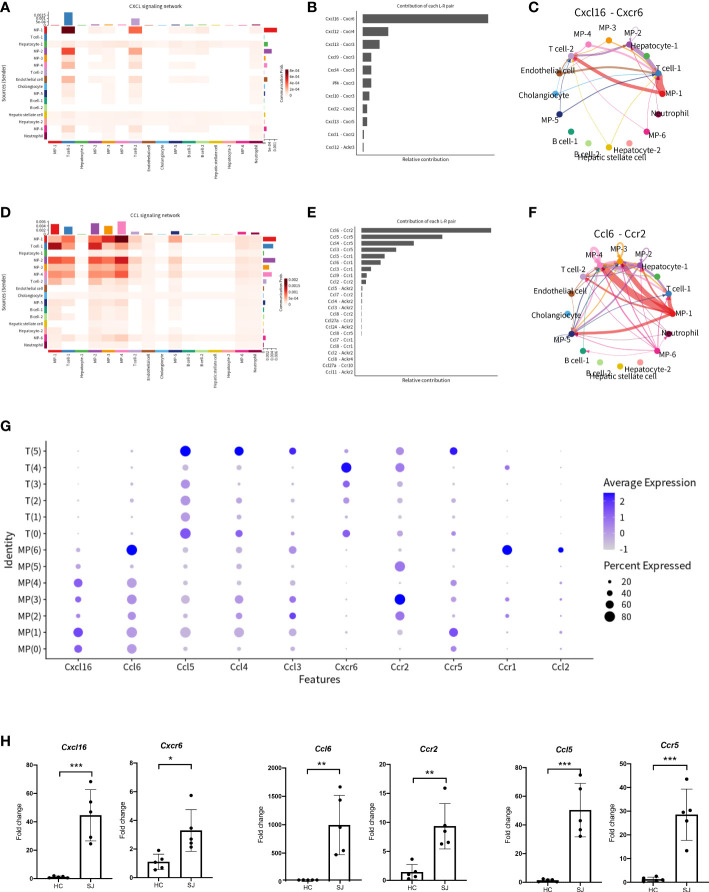
CXCL and CCL signaling pathway network in fibrotic liver of schistosomiasis japonica. **(A)** Heatmaps of the differential number of interactions between different cell clusters in CXCL signaling network. **(B)** Relative contribution of each ligand-receptor pair to CXCL signaling network. **(C)** Circle plots displaying the Cxcl16-Cxcr6 network between different cell clusters. **(D)** Heatmaps of the differential number of interactions between different cell clusters in CCL signaling network. **(E)** Relative contribution of each ligand-receptor pair to CCL signaling network. **(F)** Circle plots displaying the Ccl6-Ccr2 network between different cell clusters. **(G)** Bubble plots of different chemokines and their receptor in MPs and T cells. **(H)** The qPCR of Cxcl16, Cxcr6, Ccl6, Ccr2, Ccl5 and Ccr5 in schistosome-fibrotic livers(n=5) and normal livers(n=5). The significance level was tested by unpaired t test and the data are shown as the mean ± s.d value (*p < 0.05; **p < 0.01; ***p < 0.001; ****p < 0.0001).

Infiltration of cells promoted formation of granulomas and hepatic fibrosis in schistosomiasis. To better understand cell-to-cell communications, we explored CXCL and CCL signaling pathway with CellChat. The aggregated cell-cell communication network was shown in **Figure 5A** among different cell clusters about CXCL signaling pathway. Contribution of each L−R pair showed that Cxcl16-Cxcr6 were the most dominant L−R among CXCL signaling pathway and circle plot showed interaction of Cxcl16-Cxcr6 among all clusters (**Figure 5B, C**). Intricate communication network of different cell clusters was also analyzed for CCL signaling pathway (**Figure 5D**). Ccl6-Ccr2 and Ccl5-Ccr5 were the main contributor of L−R for CCL signaling pathway (**Figure 5E**). Circle plots showed the communication of Ccl6-Ccr2 among different cell cluster(**Figure 5F**). The bubble plots indicated a close relationship between T cells and MPs *via* CXCL and CCL signaling pathway. We further explored the expression of main chemokines and chemokine receptors on T cells and MPs. *CCL6* was mainly expressed in MP(6) while T(5) had high expression of *CCL3*, *CCL4* and *CCL5*. *Cxcr6* was mainly showed in T(4), while *Ccr2* was mainly expressed in MP(3). *Ccr5* was predominantly expressed in MP(1) and T(5) (**Figure 5G**). Thus, Cxcl16-Cxcr6, Ccl6-Ccr2 and Ccl5-Ccr5 were the most dominant L−R, which were significantly increased in fibrotic livers when compared with normal livers (**Figure 5H**).

### FASLG signaling pathway in fibrotic liver of *Schistosoma japonica*

**Figure 6:**
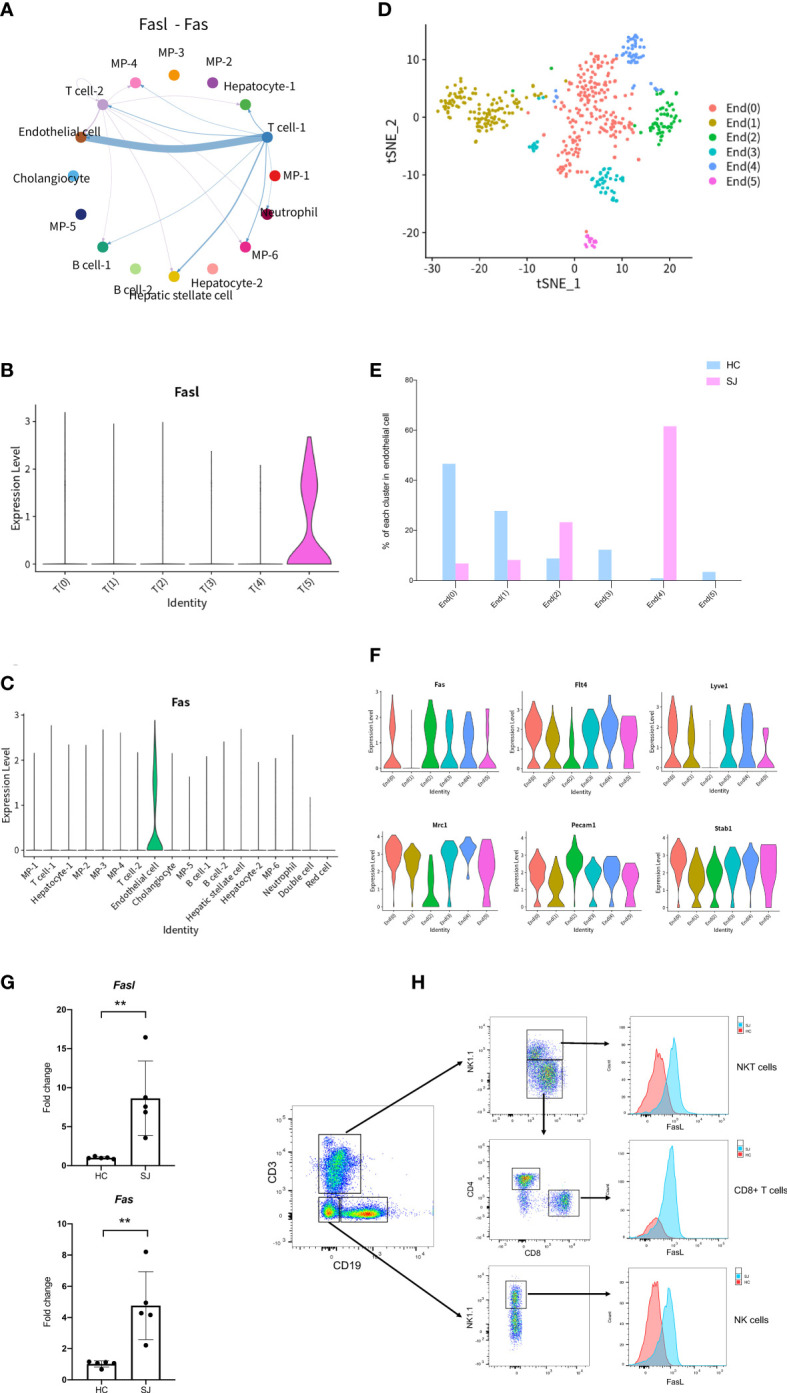
FASLG signaling pathway in fibrotic liver of schistosomiasis japonica. **(A)** Circle plots displaying the Fasl-Fas signaling network between different cell clusters. **(B)** Violin plots of Fasl in each T cells cluster. **(C)** Violin plots of Fas in different cells cluster. **(D)** tSNE plots of endothelial cells for six clusters. **(E)** The percentage change tendency of each endothelial cells cluster in two groups. **(F)** Violin plots of genes in each endothelial cells cluster. **(G)** The qPCR of Fasl and Fas in schistosome-fibrotic livers(n=5) and normal livers(n=5). The significance level was tested by unpaired t test and the data are shown as the mean ± s.d value (*p < 0.05; **p < 0.01; ***p < 0.001; ****p < 0.0001). **(H)** The expression of FasL in NKT cells, CD8+ T cells and NK cells. Gating strategy for the identification of T cells (CD3+CD19-NK1.1-), B cells(CD19+CD3-NK1.1-), NK cells(NK1.1+CD3-CD19-), NKT cells(CD3+NK1.1+), CD4+ T cells(CD3+CD4+NK1.1-) and CD8+ T cells(CD3+CD8+NK1.1-) (gated on live cells and CD45+ cells).

To better understand mechanisms of hepatic fibrosis of schistosomiasis, CellChat was used to further analyze cell-to-cell signaling and interaction among different cluster. Results showed that FASLG signaling pathway (Fasl-Fas) was closely associated with T cell and endothelial cell (**Figure 6A**). *Fasl* was expressed predominantly in T(5), which was only shown in the SJ group (**Figure 6B**). *Fas* was mainly expressed in endothelial cells (**Figure 6C**). tSNE plot showed endothelial cells were grouped into 6 clusters (**Figure 6D**). The SJ group was mainly consisted of End(2) and End(4), while the HC group was mainly composed of End(0) and End(1) (**Figure 6E**). *Fas* was present in End(0), End(2), End(3), End(4) and End(5), which expressed the marker genes of liver sinusoidal endothelial cells (LSECs)(Flt4, Mrc1,Pecam1and Stab1) (**Figure 6F**) (31). The qPCR analysis indicated Fasl and Fas were significantly increased in fibrotic livers (**Figure 6G**). The expression of Fasl in NKT cells, CD8+ T cells and NK cells were higher in fibrotic livers than that in normal livers (**Figure 6H**).

## Discussion

Hepatic fibrosis of schistosomiasis impacting quality of life and survival is a poor outcome of patients, which is often accompanied with portal hypertension, ascites, splenomegaly and gastro-esophageal variceal bleeding (32). Eggs deposited in the liver are pathogenic factors of schistosomiasis, which induce granuloma formation and hepatic fibrosis. However, there is no effective measure to prevent and treat schistosome-induced hepatic fibrosis. In order to improve therapy of hepatic fibrosis in schistosomiasis, the mechanisms of hepatic fibrosis in schistosomiasis require further investigation. Single-cell sequencing, a booming emerging technology, reveals an increasing number of biological insights at the single-cell resolution (33). We applied single-cell sequencing to explore the mechanism of hepatic fibrosis in murine schistosomiasis *japonica*. A better understanding of pathogenesis could shed new light for treatment of hepatic fibrosis in schistosomiasis.

Single-cell sequencing was performed on mouse fibrotic liver after *S. japonicum* infection and normal mouse liver. After conducted quality control analyses, a total of 10,403 cells, which grouped into 18 major cell clusters, were eventually included in our analysis. There was obvious difference between the SJ group and the HC group for different cell clusters. T cell-1(30.12%), MP-2(19.76%) and MP-4(15.44%) were predominantly shown in the SJ group, while MP-1(32.86%), MP-3(12.00%) and Hepatocyte-1(14.94%) were mainly presented in the HC group. Eggs trapped in liver are inducers of immune responses and recruitment of variable immune cells (34). It was obvious that massive T cells infiltrated into mouse fibrotic liver after *S. japonicum* infection, which indicated T cells are tightly linked to development of schistosome‐associated liver fibrosis.

We further performed cluster analysis on different cells separately to discover specific cell subsets for schistosome‐associated liver fibrosis. All of T cells were clustered into 6 main clusters, while T(4) and T(5) were only existed in the SJ group. T(4) were Th2 cells with marker genes of *Cd4*, *Gata3* and *Il4*. T(5) had high expression of *Prf1* and low expression of *Cd8a*, which were NKT cells. Previous research showed that eggs could strongly invoke Th2 response and IL-13, a Th2 cytokines, directly promoted severe liver fibrosis (35, 36). During murine schistosomiasis, NKT cells could recognize glycolipids presented by CD1d on APCs, inducing the production of type 2 cytokines (IL-4, IL-5, IL-13) and influence the Th1/Th2 balance of the immune response (37–39). Our results showed that there was close communication between T cell and FASLG signaling pathway. NKT cells had high expression of *Fasl* while LSECs had high expression of *Fas*. The expression of Fasl in NKT cells, CD8+ T cell and NK cell were significant higher in schistosome‐associated liver fibrosis than normal livers. Apart from its apoptotic function of interaction of Fas with its ligand FasL, FasL has also been reported to induce the production of numerous proinflammatory cytokines. Meanwhile, soluble FasL fragment (sFasL) cleaved by metalloproteinases could promote cell proliferation and has little to no capacity to induce apoptosis (40). The signaling pathways may act through DAX, RIP, SUMO, FAF-1, FAP-1, and others. However, further study was needed to investigate the mechanisms of FasL in schistosome‐associated liver fibrosis. KEGG pathway analysis was performed and the DEGs between NKT cells and other T cells subsets were involved in natural killer cell-mediated cytotoxicity and chemokine signaling pathway. We collected all mononuclear phagocytes which were grouped into 7 clusters. The SJ group was mainly consisted of MP(1) and MP(3). MP(1) with *Cd163*, *Marco*, *Timd4* and *Adgre1* were the Kupffer cells, while Ccr2+CD163-MARCO-TIMD4-MP(3) might be macrophage which migrate into liver (17). Macrophages are broadly divided into classically activated macrophages (M1) and alternatively activated macrophages (M2). MP(1) also had high expression of *Arg1*, *Retnla* and *Chil3*, marker genes of M2. Our results indicated that Kupffer cells might undergo M2-like polarization in fibrotic liver of schistosomiasis. The research showed that Th2-type cytokines (IL-4 and IL-13) respond to inflammatory reactions for egg and promote M2 polarization (34). The KEGG pathways about the DEGs between MP(1) and other MP subsets were related to NOD-like receptor signaling pathway, lysosome and phagosome. B(0) and B(1) were mainly showed in the SJ group and the SJ group was mainly composed of B(0) and B(1). *Ighm* and *Ighd* were expressed in B(0), which were naive B cells. B(3) were plasmablast with high expression of *Prdm1*, *Sdc1* and *Irf4*. The enriched KEGG pathways about the DEGs between B(1) and other B cell cluster were related with protein processing in endoplasmic reticulum and protein export. *Mki67* was also presented in B(1). The role of B cells requires further study about hepatic fibrosis in schistosomiasis. Cellular infiltration at liver relates to the progression of liver fibrosis in schistosomiasis. CXCL and CCL signaling pathway in CellChat showed that Cxcl16-Cxcr6, Ccl6-Ccr2 and Ccl5-Ccr5 were the most dominant L−R and there were close communications between T cells and MPs. Kupffer cells(MP(0), MP(1) and MP(4)) showed high expression of Cxcl16, while the receptor Cxcr6 were mainly expressed in T(4)(Th2 cell). Thus, Kupffer cells might have potential in recruiting Th2 cells *via* the Cxcl16-Cxcr6 signaling axis. Ccl6 can also be expressed by macrophages, particularly MP(6). Ccr2, a receptor for Ccl6, was also not only expressed in MP(3) of the SJ group, but also in T(4)(Th2 cell) and T(5)(NKT cell), a higher level of expression of Ccr2 than other T cells. Meanwhile, T(5) (NKT cell) had high expression of CCL3, CCL4 and CCL5, which might recruit T(5) (NKT cell) and MP(1) *via* interaction with Ccr5. Our results initially showed a tight crosstalk between T cells and macrophages in liver fibrosis of schistosomiasis. These results indicated that Cxcl16-Cxcr6, Ccl6-Ccr2 and Ccl5-Ccr5 might play a role in the pathogenesis of schistosomiasis associated liver fibrosis and interrupting those signaling pathways could help to alleviate hepatic fibrosis.

Small sample size and absence of liver perfusion is shortcomings of this study. However, we used perfused livers samples to validate the results from sc-RNA sequencing analysis, including q-PCR and flow cytometry analysis. Our research preliminarily profiled a immunological network of hepatic fibrosis in murine schistosomiasis japonica, which might contribute to a better understanding of the mechanisms of liver fibrosis in schistosomiasis. However, further study is needed to explore the role of FASLG signaling pathway and CXCL and CCL signaling pathway(Cxcl16-Cxcr6, Ccl6-Ccr2 and Ccl5-Ccr5) in liver fibrosis of schistosomiasis.

## Data availability statement

The original contributions presented in the study are publicly available. This data can be found in the GEO database, accession number: GSE220286.

## Ethics statement

The animal study was reviewed and approved by the Ethics Committee of the 3rd Xiangya Hospital of Central South University.

## Author contributions

YZ, JL and YM contributed to the study design. YZ, JJ, CG, ZZ performed experiments. YZ, HL and CZ contributed to analysis of data. YZ, JL, YM contributed to the manuscript development. JL and YM acquired funding for the study. All authors contributed to the article and approved the submitted version.
